# Prognostic humility and ethical dilemmas after severe brain injury: Summary, recommendations, and qualitative analysis of Curing Coma Campaign virtual event proceedings

**DOI:** 10.3389/fnhum.2023.1128656

**Published:** 2023-03-31

**Authors:** Natalie Kreitzer, Brooke Murtaugh, Claire Creutzfeldt, Joseph J. Fins, Geoff Manley, Aarti Sarwal, Neha Dangayach

**Affiliations:** ^1^Department of Emergency Medicine, University of Cincinnati, Cincinnati, OH, United States; ^2^Brain Injury Program Manager, Department of Rehabilitation Programs, Madonna Rehabilitation Hospital, Lincoln, NE, United States; ^3^Department of Neurology, UW Medicine, Seattle, WA, United States; ^4^Division of Medical Ethics, Weill Cornell Medicine, New York, NY, United States; ^5^Yale Law School, New Haven, CT, United States; ^6^Department of Neurological Surgery, University of California, San Francisco, San Francisco, CA, United States; ^7^Department of Neurology, Wake Forest University, Winston-Salem, NC, United States; ^8^Departments of Neurosurgery and Neurology, Icahn School of Medicine at Mount Sinai, New York, NY, United States

**Keywords:** severe brain injury, caregiver, comfort care, prognosis, disorders of consciousness

## Abstract

**Background:**

Patients with severe acute brain injuries (SABI) are at risk of living with long-term disability, frequent medical complications and high rates of mortality. Determining an individual patient’s prognosis and conveying this to family members/caregivers can be challenging. We conducted a webinar with experts in neurosurgery, neurocritical care, neuro-palliative care, neuro-ethics, and rehabilitation as part of the Curing Coma Campaign, which is supported by the Neurocritical Care Society. The webinar discussed topics focused on prognostic uncertainty, communicating prognosis to family members/caregivers, gaps within healthcare systems, and research infrastructure as it relates to patients experiencing SABI. The purpose of this manuscript is to describe the themes that emerged from this virtual discussion.

**Methods:**

A qualitative analysis of a webinar “Prognostic Humility and Ethical Dilemmas in Acute Brain Injury” was organized as part of the Neurocritical Care Society’s Curing Coma Campaign. A multidisciplinary group of experts was invited as speakers and moderators of the webinar. The content of the webinar was transcribed verbatim. Two qualitative researchers (NK and BM) read and re-read the transcription, and familiarized themselves with the text. The two coders developed and agreed on a code book, independently coded the transcript, and discussed any discrepancies. The transcript was analyzed using inductive thematic analysis of codes and themes that emerged within the expert discussion.

**Results:**

We coded 168 qualitative excerpts within the transcript. Two main themes were discussed: (1) the concept of prognostic uncertainty in the acute setting, and (2) lack of access to and evidence for quality rehabilitation and specialized continuum of care efforts specific to coma research. Within these two main themes, we found 5 sub-themes, which were broken down into 23 unique codes. The most frequently described code was the need for clinicians to acknowledge our own uncertainties when we discuss prognosis with families, which was mentioned 13 times during the webinar. Several strategies were described for speaking with surrogates of patients who have had a severe brain injury resulting in SABI. We also identified important gaps in the United States health system and in research to improve the care of patients with severe brain injuries.

**Conclusion:**

As a result of this webinar and expert discussion, authors identified and analyzed themes related to prognostic uncertainty with SABI. Recommendations were outlined for clinicians who engage with surrogates of patients with SABI to foster informed decisions for their loved one. Finally, recommendations for changes in healthcare systems and research support are provided in order to continue to propel SABI science forward to improve future prognostic certainty.

## Introduction

Approximately 258 per 1,00,000 patients per year in the United States sustain a severe acute brain injury (SABI), including traumatic and non-traumatic etiologies (Kondziella et al., 2022). Patients with severe neurologic insults such as these have the highest rates of long-term disability when compared to any other disease process (Murray et al., 2013; Gooch et al., 2017; WHO, n.d.). When SABI occurs, decisions of whether life sustaining measures should be maintained or discontinued are often left to surrogate decision makers such as family members/caregivers, a durable power of attorney or a guardian (Keating et al., 2010; Barclay et al., 2011; Fins, 2015). In order for surrogates to make treatment decisions on a patient’s behalf, they must understand the diagnosis and prognosis as it relates to SABI from the patient’s healthcare team. However, it is difficult to predict which patients will have long term severe disability, which patients may achieve functional improvement and which ones will be able to adapt to a new health state and regain a good quality of life. In addition, it may be important to consider the patient’s support system and environment and consider how well they will adapt and or be able to support their loved one with a new level of disability (Wilson and Gilbert, 2008; Creutzfeldt and Holloway, 2012). In most cases of SABI, patients have not previously provided written, explicit wishes for continued care within an advance directive (AD) (Alonso et al., 2017; Sutter et al., 2020; Rutz Voumard et al., 2021). Thus, families are often left to assume the responsibility of making life or death treatment decisions for their loved one (Thompson et al., 2003; Adelman and Zahuranec, 2012; Sutter et al., 2020). Although many patients with SABI improve significantly over months and years post-injury, early mortality is high, and most patients who die do so after a decision to withdraw life-sustaining treatments (Zahuranec et al., 2010; Turgeon et al., 2011; Kowalski et al., 2021). Because of the complexities related to prognostic uncertainty, the term “prognostic humility” has been used to describe gaps in knowledge, understanding, and communication of prognostic uncertainty after SABI (Fins, 2020, 2022).

In order to better understand issues related to prognosis, family/caregiver engagement and systems of care for patients with SABIs, we conducted a webinar through the Neurocritical Care Society’s Curing Coma Campaign (Supplementary Table 1). The Curing Coma Campaign began in 2019, and is a “public health initiative designed to develop and implement coma treatment strategies that improve human lives” (Curing Coma, n.d.). The Community of Collaborators (CoC) is a module of the Curing Coma Campaign with the goal of discussing issues that are pertinent to families, caregiving, and follow-up. This webinar was the first in a series of planned webinars designed to integrate the discussion of numerous aspects of caregiving after a SABI. The purpose of the webinar and subsequent qualitative analysis was to obtain qualitative responses from experts in the fields of neurosurgery, neurocritical care, palliative care, ethics, and rehabilitation in a virtual focus group environment. The authors describe the major themes and discussion points of this educational webinar. We report the findings that emerged from this expert discussion, which include best practice recommendations to clinicians who are speaking to surrogates of patients with SABI, suggestions for change in healthcare systems to support SABI survivors across the continuum of care, and important gaps in research to improve SABI care.

## Materials and methods

This manuscript is a qualitative analysis of a webinar “Prognostic Humility and Ethical Dilemmas in Acute Brain Injury” that took place as part of the Neurocritical Care Society’s Curing Coma Campaign on September 28, 2021, and was aired on October 5, 2021.

This webinar was designed by the Curing Coma Campaign “Community of Collaborators” module as a panel discussion between known experts in the field, all included as authors, with two moderators presenting open ended structured questions with no formal presentations. A multidisciplinary group of experts were invited as speakers and moderators of the webinar. Content experts involved in the webinar represented the fields of adult neurosurgery, Neuroethics, rehabilitation, neurocritical care, and neuro-palliative care from throughout the United States (Supplementary Table 2). The moderators (NK and BM) were trained in Neurocritical Care and Neurological Rehabilitation. Panel questions to facilitate coma prognostic discussion were developed by the core members of the CoC, and the final draft of questions were approved by the group. All questions were designed to be open-ended, and to spark a dialogue among all members of the group. Follow up questions were developed to go in depth on certain topics. It was anticipated that the discussion amongst the group would bring out rich, unplanned commentary. The webinar was recorded through Zoom© virtual platform and transcribed verbatim.

### Analysis

The content of the transcript was analyzed using inductive thematic analysis after the recorded webinar was listened to, transcripts were made, read, re-read, and the coders had familiarized themselves with the text. Inductive analysis is a data-driven process of coding the data without attempting to fit it into an existing coding framework or the researcher’s own analytic preconceptions (Braun and Clarke, 2006). All discussion components mentioned by participating speakers and moderators were included in the analysis. A list of themes and codes identified through this discussion from the group of content experts were initially developed, with a code book describing the definition of each code. This was edited multiple times by two investigators (BM and NK), and a final code book was agreed upon. The transcript was coded by the two investigators, and codes were discussed among the two authors to resolve disagreements. In the case that the final codes and themes could not be resolved by discussion among these two authors, a third author would have been appointed to resolve any discrepancies. Results were brought back to the author (and presenter) group, and their feedback solicited as a “member check” (Taylor and Bogdan, 1998).

## Results

The webinar was aired on October 5, 2021. It was 1 h in length, followed by a 30-min Q and A session. Two main themes were identified: (1) the concept of prognostic uncertainty, and (2) lack of access to and evidence for quality rehabilitation and specialized continuum of care efforts specific to coma research. Within these two main themes, we identified 5 sub-themes, which we further broke down into 23 unique codes (Table 1). We coded 168 total unique excerpts from the discussion. The two coders (NK and BM) initially agreed on 137/(82%) of codes after independent review of the transcript, prior to discussion. After discussion, there were no discrepancies between the two coders. The most commonly coded transcript extracts fell into the codes of “healthcare systems do not exist or access to care is limited” (*n* = 21), describing issues with long-term rehabilitation not existing or not being available to patients in all geographic areas of the United States; “biases” (*n* = 19) which describes conscious and unconscious biases such as nihilism, the self-fulfilling prophecy, disability biases, or other biases, and ways clinicians should evaluated our own biases; and “acknowledging our own uncertainty” (*n* = 14), which describes uncertainties that clinicians have with prognosis (Supplementary Table 2).

**TABLE 1:** Theme, sub-theme, and coding structure.

Codes	Description/Definition of codes	Number of times code was used
**Theme 1: Prognostic uncertainty**		
**Subtheme 1: Problems with prognosis that we need to understand as clinicians/researchers**		
Language, communication, and health literacy	Clinicians have inconsistency among ourselves with the language and terminology we use. Families may not understand medical language. “*One of the things I think we have to be familiar with, and recognize is the language we use. Coma, vegetative state, minimally conscious state. You know, even stroke. People don’t know what we’re talking about*,” “*If there are inconsistencies and a lack of consensus within the medical field, how do we even begin to help families understand if we’re even struggling to purely understand and define this population*.”	6
Only have limited information	The patient is generally in the early time course, so information is often limited. “*We are forced to make all these moral choices before we have all the facts.*”	4
Patient variability	Each patient is unique in terms of prognosis, making brain injury prognostication challenging: “*This is not a stereotypic disease. Everyone has their own injury. And everyone is going to behave differently and they’re going to have comorbidities. And there’s a lot of uncertainty*.”	4
Biases	There are many biases clinicians may have. These include nihilism, self-fulfilling prophecy, disability bias, and others. Clinicians have conscious and unconscious biases that we should evaluate within ourselves so that we can improve. *“For a long time in neurocritical care, it seems we have been riddled with nihilism.”*	19
Culture	Describes the cultural ways clinicians think about unconsciousness and the right to die after brain injury. “*We as clinicians have a very different view of what a good outcome is, versus what a family has as a good outcome. And we need to spend more time talking to families about what their perspective of outcome is*.”	7
**Subtheme 2: Communication strategies to help with uncertainty as we are communicating to families**		
Information changes	It is important to convey to families that information about prognosis changes over time after a brain injury. This information may pertain to comorbidities and their interplay with the patient’s initial brain injury. This may also involve proving psychological support/counseling for families to manage changing expectations. *And (decisions) are going to need to be made with information that is going to change and evolve even over a patient’s time with us as well as while they are in a rehabilitation setting, or continuing to recover in a long-term care facility*	4
Need for clinical caregiver partnership	Clinicians need to see ourselves as partners with patients and surrogates. The partnership should be open, transparent, and clinicians should recognize that family/caregivers knows the patient as a human. “*I often tell families that I might be an expert in what I’m doing, but you are the expert in the person, the human being we are seeing in front of us.*”	6
Trajectories	Approach prognosis from the stance of trajectories, milestones, and a positive uncertainty. Describe short- and long-term milestones. Recognize uncertainty decreases over time. This information pertains to individual trajectories of acute brain injury. *“We talk about the cone of uncertainty, and how it gets narrower and narrower over time.”*	13
New normal	Talk to families about what a new normal is like after severe brain injury. Psychological support/counseling regarding preparing for the new challenges expected through short- and long-term care of SCH patients. *“(It’s important to) prepare the loved ones, the family members, for this new state of normal. It’s not going to be who this person was before they became our patient. It’s not going to be different; it’s not going to be bad, or it’s not going to be better, it’s just going to be different.”*	3
Certainty	Explain to families the things we can be certain about. “*I do think there are some times, certain things we can be relatively certain about. As an example, I am certain that this is going to be a very long road*.”	3
**Sub-theme 3: Strategies in managing ourselves as healthcare providers in the face of uncertainty**		
Acknowledge our own uncertainty	Recognize that we have uncertainties ourselves, that this job is a humbling responsibility. “*I’ve seen enough people that I thought would do well that didn’t and people that I thought would do terrible that didn’t, that I really stopped prognosticating. So, I think it’s a delicate walk that we do with families*.”	14
Making peace and learning	We need to make peace with decisions and continue learning more. “*When we go back to the same decision, a year or more, we’re probably going to make a different decision. So, we have to make our peace with that*.”	3
Incorporate science	Clinicians must incorporate scientific discoveries into our practice. Recognize that in many cases, early withdrawal of care is inconsistent with science. “*There is a remarkable emerging literature about how injured brains recapitulate the developmental process with sprouting and pruning of axons.”*	10
**Theme 2: Systems approach: Healthcare and research issues**		
**Sub theme 1: Absence of, or too little support from health systems**		
Healthcare systems do not exist or access to care is limited	The system of care necessary to help patients with severe brain injuries is absent. It is hard to partner with patients and families when many features such as long-term rehabilitation do not exist everywhere, since even when some systems exist, they are not available to everyone. “*If you live in the middle of the country, where are you going to go? There’s nowhere to go. There’s no heart in the heartland*.”	21
Fiscal Issues	There is too little of a fiscal investment in severe brain injuries. Caring for these patients requires years of expensive care that is unmet. “W*e make a moral and fiscal investment in these people, and if we aren’t going to pay for the tail of the injury, the first 6 weeks, and the next 20 years probably cost the same thing*.”	6
Lacking coma science	More research is needed, more dissemination of research is needed. “*In these diseases (cancer) where we have so much more data and we can understand outcomes better, and we have a lot more biomarkers to know where we are on that course and that journey*.”	4
Siloed by diagnosis	Patients are siloed based on diagnosis, rather than functional status, and this may get in the way of rehabilitation. Health systems focused on patients with similar spectrum of disability despite having a different etiology might benefit from shared resources. e.g., post-acute care rehabilitation tends to focus on ischemic stroke or traumatic brain injury. *“We have to be less obsessed with the diagnosis and more concerned with the functional status.”*	5
**Sub-theme 2: Solutions to healthcare or research related issues**		
Agree on outcomes and timing	We need to define outcomes as a neurological care continuum, and determine when outcomes should be assessed. Current outcomes research and healthcare systems are heterogeneous in terms of timeline and outcome definition. *“But brains recover by biologic mechanisms, not reimbursement criteria.”*	10
Optimize short term care	We should provide optimal care in the short term to prevent common complications of brain injuries. Short term care targets should not be biased or held back by prognostication discussions. *“I think it’s important that we don’t cause iatrogenic deaths either through the avoidable urinary tract infection and bed sore or pneumonia.”*	2
Education interventions that target informed families	Systems must be in place that appropriately inform families, such that withdrawal of care is only considered after properly informing a family/caregivers. Such level of being informed should be considered at the same level as informed consent. *“Speaking of the goals and wishes for the patient who can’t speak for themselves. If it’s a life well lived, and they would not want to take that chance to see what would happen next, in that moment in time, our role as being those facilitators of a dignified death is also as important as being a facilitator of a dignified life.”*	8
Civil rights	We may want to look at prognosis after severe brain injury through a civil rights violation lens. Civil rights, guarantees of equal social opportunities and equal protection under the law, regardless of race, religion, or other personal characteristics. *“This is not just an economic thing. This is about civil rights. This is about the rights of people who have severe brain injury to fully engage in society.”*	4
Team	Systems should assure that a healthcare team should be on the same page and supportive of one another when discussing with families. *“The most important thing we do before we meet with families is to have the pre-meeting huddle. We don’t share our confusion and our conflict. We try to reach a consensus about where this patient is.”*	6
Need for research	There is a need for continued research into coma science, entailing precise outcomes, better prognostication tools, and an understanding of the timeline for patients with severe acute brain injuries. *“And so we need to kind of learn more about the biology to harmonize the financing to go with the science.”*	6

Heat map color configuration: 1–5: orange; 6–10: aqua; 11–15: purple; 15–19: green; 20, and over: red.

### Theme 1: Prognostic uncertainty

#### Sub-theme 1: Problems with prognosis that we need to understand as clinicians/researchers

Several challenges were discussed related to prognostic discussions within groups of interdisciplinary professionals and between interdisciplinary healthcare professionals and surrogates. These included the type of communication and language used by healthcare providers, which may not meet the healthcare literacy needs of individual families/caregivers. In many cases, healthcare professionals may not be using consistent language themselves to describe SABI or coma, which further complicates discussions with surrogates. Further, organizations such as the American Medical Association have called for increased awareness of patient literacy when discussing healthcare matters (Ad Hoc Committee on Health Literacy, 1999). These issues were highlighted with the quote: “*One of the things I think we have to be familiar with, and recognize is the language we use. Coma, vegetative state, minimally conscious state. You know, even stroke. People don’t know what we’re talking about*.”

Biases that influence prognostication such as nihilism defined as skepticism of treatment, (Merriam-Webster, n.d.) the self-fulfilling prophecy, defined as “an erroneous belief or expectation that leads to its fulfillment,” (Merton, 1948) the disability paradox, defined as those patients with disabilities who report a good quality of life despite the fact that those externally may report an imagined poorer quality of life (Albrecht and Devlieger, 1999; Ubel et al., 2005), or other biases were brought up as an issue that arises in discussions with surrogates. It is important to recognize our own ingrained cultural beliefs and how they may differ from surrogates’ cultural beliefs related to treatment preferences and decision-making. “*We as clinicians have a very different view of what a good outcome is, versus what a family has as a good outcome. And we need to spend more time talking to families about what their perspective of outcome is*.”

#### Sub-theme 2: Communication strategies to help with uncertainty

The second sub-theme focused on communication strategies to help with uncertainty as clinicians speak to surrogates about prognosis after SABI. Several strategies were discussed that may be beneficial. The healthcare team should ideally view surrogates as partners. As such, it is important to clearly convey to surrogates early after SABI that prognostic information changes over time. Although some aspects of prognoses after SABI may be uncertain, healthcare providers should highlight to surrogates the aspects of their care and prognosis that are more certain. In doing so, it is important to also describe to surrogates that the prognostic trajectory after SABI may become clearer over time in some cases, and that this may improve the ability to better understand prognosis. This trajectory can be augmented with short- and long-term milestones expected for the patient. Since the trajectory of many patients with SABI in prior literature is unknown due to withdraw of life sustaining treatments, serial monitoring and communication of an individual patient’s trajectory may provide useful information to families/caregivers in understanding prognosis (Hammond et al., 2021). This serial monitoring requires the use of multidisciplinary professionals to work cohesively together along the continuum of care. One speaker said “*And (decisions) are going to need to be made with information that is going to change and evolve even over a patient’s time with us as well as while they are in a rehabilitation setting, or continuing to recover in a long-term care facility.”*

#### Sub-theme 3: Strategies that clinicians can use to educate ourselves in the face of uncertainty

The third sub-theme described strategies clinicians can use to educate ourselves and improve our ability to care for surrogates of patients who face prognostic uncertainty. It was discussed that over time, it is important that healthcare providers reflect on past decisions, learn the emerging science in SABI prognosis, and make peace with prior decisions, so that we can better care for patients in the future. The most commonly discussed concept related to this was the need for us to acknowledge our own uncertainties about prognosis at times. One speaker said “*I’ve seen enough people that I thought would do well that didn’t and people that I thought would do terrible that didn’t, that I really stopped prognosticating. So, I think it’s a delicate walk that we do with families*.”

### Theme 2: A systems approach to healthcare and research issues

#### Sub-theme 4: Absence of, or too little support in healthcare systems

The fourth sub-theme describes the phenomenon that there are problems when caring for patients with SABIs in the United States related to the lack of resources within healthcare systems, rehabilitation facilities, and outpatient and community-based services. This may mean that systems do not exist, may be geographically sparse such that they are not widely available to a large portion of the United States population, or that ongoing care and rehabilitation needs are fiscally unattainable for many SABI survivors. The panel mentioned that patients may receive excellent emergency stabilization and acute care, only for funding in post-acute care to be lacking. Oyesanya et al. (2021) previously described this phenomenon in a Medicare database study in which they noted significant differences in rehabilitation outcomes following TBI based on geographical location within the United States. In addition, more research is needed to best understand how to optimize patients’ rehabilitation needs. These issues were highlighted with the quote, “*If you live in the middle of the country, where are you going to go? There’s nowhere to go. There’s no heart in the heartland*.”

#### Sub-theme 5: Solutions to healthcare and research related issues

The final sub-theme described strategies for improvements within healthcare systems and within coma research. In addition to optimizing short term care and preventing complications in the acute period after SABI, it is important that healthcare systems have plans in place to make sure families are well-informed during this time. The utilization of a team-based approach to coma care can facilitate this. One example of a way to make sure families are well-informed and have not heard differing messages from members of the care team was to institute a “huddle,” or a discussion within the healthcare team prior to meeting with family or caregivers (Hammond et al., 2021).

Lastly, we discussed the critical need for more research and dissemination of coma science in general. This encompasses a need for the scientific community studying coma outcomes to agree on the types of outcomes and timing after SABI of when to measure these outcomes. This was highlighted with the quote, “*there needs to be a plug for funding more research. Because we don’t know. We need to say what we do know, and we realize what we don’t know. We need to explore people’s values knowing how well they are going to recover and adapt in the future. There’s just so much we need to learn and systematically research*.”

## Discussion

This qualitative study highlighted numerous issues related to prognostic uncertainty and healthcare systems after a SABI. There are numerous challenges related to prognostication of patients with SABI, particularly during the early ICU course of the injury. Although many patients with SABI may regain consciousness, functional independence, and even experience late improvements in outcomes, many others may not do well or would not want to live with SABI, making prognostication challenging (Whyte et al., 2013; Giacino et al., 2020; Kowalski et al., 2021). The panel discussed important solutions such as identifying our own biases as clinicians that lead to premature withdrawal of life-sustaining treatment, such as nihilism, the self-fulfilling prophecy, or the disability paradox. Additionally, the panel discussed that the family or caregivers may have inaccurate pre-conceived notions that withdrawal of life sustaining treatment may not be possible after the ICU course of illness, sometimes described as a “missed opportunity (Cochrane, 2009).”

This webinar focused extensively on discussions of prognostic uncertainty between clinicians and family surrogate decision makers. Some solutions have been described previously in published literature, such as assuring that clinicians understand current evidence as well as gaps in research related to coma science before conducting education and counseling with families. Clinicians need to be intentional to update discussions with surrogates using advanced tools, tailored predictions and meaningful long term endpoints to portray an accurate prognosis (Hammond et al., 2021). Family or caregiver discussions should specify both the predictions and level of confidence in predictions (Hammond et al., 2021). The disorders of consciousness practice recommendations describe best practices for counseling families about prognosis, and recommends that clinicians avoid statements that indicate a universally poor prognosis in the first days and weeks post-injury (Giacino et al., 2018). A recent NIH workshop discussed recommendations similar to those that were brought up in our webinar, which included, (1) ways to communicate more clearly and consistently, (2) better assistance with navigating resources and access to places for families to care for themselves, and (3) opportunities for families to remain connected with their loved ones, social support networks and clinical team (Muehlschlegel et al., 2022). New solutions for discussions with surrogates focusing on prognosis were identified in the webinar. One example was the suggestion to approach prognosis as a trajectory, with short- and long-term milestones. As more time passes, the level of uncertainty may decrease, and surrogates may gain a better understanding of prognosis.

Although numerous problems related to prognosis and the healthcare system after SABIs were discussed, this webinar also discussed targets for improvement of care. There were two systemic issues discussed that require urgent action plans for optimizing SABI recovery. The first issue is the need for effective healthcare systems and infrastructure to care for patients who have sustained a SABI. Currently, a care continuum after SABI is not available to much of the population in the United States. The second issue is the need for more research in coma science, such as how and when to determine outcomes after SABI, and how to provide the optimum continuum of care to patients with SABI. Specifically, a unified type and time point to measure outcomes across research in SABI was deemed important by expert panelists, as well as the need for dissemination of research findings. Future work that builds from this qualitative work may investigate how the codes and themes that emerged in this work are related with one another, or even converge with one another, and how this plays a role in discovering targets for improvement of care.

Without healthcare systems support such as publicly funded long term care insurance, a budget for home health assistance, and with expensive co-pays for rehabilitation or novel treatments, and support for family caregivers, it is challenging to counsel families and to conduct research in this realm (Caplan, 2017; Sattin et al., 2014, 2017). Additionally, reports of variation in referral to rehabilitation among clinicians indicate there may be opportunities to standardize post-acute care (Swaine et al., 2018).

## Limitations

As with any small qualitative study, it is noted that findings are affected by the experience and perceptions of the participating research team as well as the composition and experiences of the participants. To limit this bias in the analysis of this webinar, we utilized expertise in qualitative methods and analysis, and we followed a systematic process. This improved the credibility and dependability of findings. An additional limitation is only two authors (NK and BM) identified and coded themes and sub-themes. Recruiting additional reviewers of the webinar transcript to identify themes, sub themes and code those themes may have identified additional salient, yet important themes related to prognostic humility. We did not have representation in the panel from neonatal or pediatric SABI, so results cannot be extrapolated into the pediatric population. A final limitation is the webinar captured the knowledge, opinions and editorials of those experts involved in the webinar. There is a wide depth and breadth of evidence and experience-informed clinicians involved in care and prognosis of patients with SABI. Other experts or family members/caregivers could have brought additional insights and views that could have impacted the identification and coding of themes within the analysis. We hope that this initial work incites further investigation using a broader survey of community members that play roles in the issues pertinent to families, caregiving, and follow-up. Further webinars exploring these perspectives are underway. We have also requested a position paper from experts to address priorities prognostic humility and ethical dilemmas with data to support those thoughts/ideas.

## Conclusion

This qualitative analysis identified and coded themes and sub-themes of an expert discussion focused on prognostic humility when approaching coma and SABI in the acute phases of care. Key themes related to acknowledging prognostic uncertainty when approaching patient care and family counseling were identified. Current approaches of prognosis as well as gaps in knowledge, comfort, and health systems create barriers to effective prognostication and support of families. It is imperative for the neurological care community to continue to engage in scientific processes to address the gaps discussed to improve prognostic discussion and advocacy for the patient with SABI and their families.

## Data availability statement

The raw data supporting the conclusions of this article will be made available by the authors, without undue reservation.

## Author contributions

CC, GM, and JF participated as expert panelists in the webinar. NK, AS, ND, and BM conducted the planning and moderating of the webinar. NK and BM drafted the manuscript and conducted the analyses. All authors revised and contributed to the manuscript.
