# Supplementary material for: Prognostic humility and ethical dilemmas after severe brain injury: Summary, recommendations, and qualitative analysis of Curing Coma Campaign virtual event proceedings
Source: Front Hum Neurosci. 2023 Mar 31;17:1128656. doi: 10.3389/fnhum.2023.1128656 (PMC10102639; doi:10.3389/fnhum.2023.1128656)
Supplement: Supplementary file 1 [file Table_1.docx]

**Supplemental Table 1: Members of Curing Coma Campaign**

| Venkatesh | Aiyagari |  | Salia | Farrokh |  | Julie | Kromm |
| --- | --- | --- | --- | --- | --- | --- | --- |
| Yama | Akbari |  | Simona | Ferioli |  | Abhay | Kumar |
| Fawaz | Al-Mufti |  | Davinia | Fernandez-Esp |  | Pedro | Kurtz |
| Sheila | Alexander |  | Ericka | Fink |  | Steven | Laureys |
| Anne | Alexandrov |  | Joseph | Fins |  | Thomas | Lawson |
| Ayham | Alkhachroum |  | Brandon | Foreman |  | Nicolas | Lejeune |
| Moshagan | Amiri |  | Jennifer | Frontera |  | Ariane | Lewis |
| Brian | Appavu |  | Rishi | Ganesan |  | John | Liang |
| Meron | Awraris Gebre |  | Ahmeneh | Ghavam |  | Geoffrey | Ling |
| Mary Kay | Bader |  | Joseph | Giacino |  | Sarah | Livesay |
| Neeraj | Badjiata |  | Christie | Gibbons |  | Andrea | Luppi |
| Ram | Balu |  | Emily | Gilmore |  | Lori | Madden |
| Megan | Barra |  | Olivia | Gosseries |  | Craig | Maddux |
| Rachel | Beekman |  | Theresa | Green |  | Dea | Mahanes |
| Ettore | Beghi |  | David | Greer |  | Shraddha | Mainali |
| Kathleen | Bell |  | Mary | Guanci |  | Nelson | Maldonado |
| Erta | Beqiri |  | Cecil | Hahn |  | Rennan | Martins Ribeiro |
| Tracey | Berlin |  | Ryan | Hakimi |  | Marcello | Massimini |
| Thomas | Bleck |  | Daniel F | Hanley |  | Stephan | Mayer |
| Yelena | Bodien |  | Jed | Hartings |  | Victoria | McCredie |
| Varina | Boerwinkle |  | Ahmed | Hassan |  | Molly | McNett |
| Melanie | Boly |  | Claude | Hemphill |  | Jorge | Mejia-Mantill |
| Alexandra | Bonnel |  | Holly | Hinson |  | David | Menon |
| Emery | Brown |  | Karen | Hirsch |  | Geert | Meyfroidt |
| Eder | Caceres |  | Sarah | Hocker |  | Julio | Mijangos |
| Elizabeth | Carroll |  | Peter | Hu |  | Dick | Moberg |
| Emilio G. | Cediel |  | Xiao | Hu |  | Asma | Moheet |
| Sherry | Chou |  | Theresa | Human |  | Erika | Molteni |
| Giuseppe | Citerio |  | David | Hwang |  | Martin | Monti |
| Jan | Classen |  | Judy | Illes |  | Chris | Morrison |
| Chad | Condie |  | Matthew | Jaffa |  | Susanne | Muehlschlegel |
| Katie | Cosmas |  | Michael L. | James |  | Brooke | Murtaugh |
| Claire | Creutzfeldt |  | Anna | Janas |  | Lionel | Naccache |
| Neha | Dangayach |  | Morgan | Jones |  | Masao | Nagayama |
| Michael | DeGeorgia |  | Emanuela | Keller |  | Emerson | Nairon |
| Caroline | Der-Nigoghoss |  | Maggie | Keogh |  | Girija | Natarajan |
| Masoom | Desai |  | Jenn | Kim |  | Virginia | Newcombe |
| Michael | Diringer |  | Keri | Kim |  | Niklas | Nielsen |
| James | Dullaway |  | Hannah | Kirsch |  | Filipa | Noronha-Falc‹ |
| Brian | Edlow |  | Matt | Kirschen |  | Paul | Nyquist |
| Ari | Ercole |  | Nerissa | Ko |  | DaiWai | Olson |
| Anna | Estraneo |  | Daniel | Kondziella |  | Marwan | Othman |
| Guido | Falcone |  | Natalie | Kreitzer |  | Adrian | Owen |
| Llewellyn | Padayachy |  | Robert | Stevens |  |  |  |
| Soojin | Park |  | Jose | Suarez |  |  |  |
| Melissa | Pergakis |  | Bethany | Sussman |  |  |  |
| Len | Polizzotto |  | Shaurya | Taran |  |  |  |
| Nader | Pouratian |  | Aurore | Thibaut |  |  |  |
| Marilyn | Price Spivack |  | Zachary | Threlkeld |  |  |  |
| Lara | Prisco |  | Lorenzo | Tinti |  |  |  |
| Javier | Provencio |  | Daniel | Toker |  |  |  |
| Louis | Puybasset |  | Michel | Torbey |  |  |  |
| Lindsay | Rasmussen |  | Stephen | Trevick |  |  |  |
| Verena | Rass |  | Alexis | Turgeon |  |  |  |
| Risa | Richardson |  | Andrew | Udy |  |  |  |
| Cassia | Righy Shinots |  | Panos | Varelas |  |  |  |
| Chiara | Robba |  | Chethan | Venkatasubba |  |  |  |
| Courtney | Robertson |  | Paul | Vespa |  |  |  |
| Benjamin | Rohaut |  | Walter | Videtta |  |  |  |
| John | Rolston |  | Henning | Voss |  |  |  |
| Mario | Rosanova |  | Ford | Vox |  |  |  |
| Eric | Rosenthal |  | Amy | Wagner |  |  |  |
| Mary Beth | Russell |  | Mark | Wainwright |  |  |  |
| Gisele | Sampaio Silva |  | John | Whyte |  |  |  |
| Leandro | Sanz |  | Briana | Witherspoon |  |  |  |
| Simone | Sarasso |  | Aleksandra | Yakhind |  |  |  |
| Aarti | Sarwal |  | Ross | Zafonte |  |  |  |
| Nicolas | Schiff |  | Darin | Zahuranec |  |  |  |
| Caroline | Schnakers |  | Chris | Zammit |  |  |  |
| David | Seder |  | Bei | Zhang |  |  |  |
| Vishank Ar | Shah |  | Wendy | Ziai |  |  |  |
| Amy | Shapiro-Rosen |  | Lara | Zimmerman |  |  |  |
| Angela | Shapshak |  | Elizabeth | Zink |  |  |  |
| Kartavya | Sharma |  |  |  |  |  |  |
| Tarek | Sharshar |  |  |  |  |  |  |
| Lori | Shutter |  |  |  |  |  |  |
| Jacobo | Sitt |  |  |  |  |  |  |
| Beth | Slomine |  |  |  |  |  |  |
| Peter | Smielewski |  |  |  |  |  |  |
| Wade | Smith |  |  |  |  |  |  |
| Emmanuel | Stamatakis |  |  |  |  |  |  |
| Alexis | Steinberg |  |  |  |  |  |  |
