# Supplementary material for: Prognostic humility and ethical dilemmas after severe brain injury: Summary, recommendations, and qualitative analysis of Curing Coma Campaign virtual event proceedings
Source: Front Hum Neurosci. 2023 Mar 31;17:1128656. doi: 10.3389/fnhum.2023.1128656 (PMC10102639; doi:10.3389/fnhum.2023.1128656)
Supplement: Supplementary file 2 [file Table_2.docx]

**Supplementary Table 2:**

**Links to Areas of Expertise**

| **Google Scholar or PubMed Links of Authors** |
| --- |
| [Kreitzer N‬](https://scholar.google.com/citations?user=plU70z8AAAAJ&hl=en&oi=ao) |
| [Murtaugh B](https://pubmed.ncbi.nlm.nih.gov/?term=Murtaugh%20B%5BAuthor%5D) |
| [Creutzfeldt CJ](https://pubmed.ncbi.nlm.nih.gov/?term=Creutzfeldt+CJ&size=200) |
| [Fins JJ](https://pubmed.ncbi.nlm.nih.gov/?term=Fins+JJ&size=200) |
| [Manley G‬](https://scholar.google.com/citations?user=0O9BbqoAAAAJ&hl=en&oi=ao) |
| [Sarwal‬ A‬](https://scholar.google.com/citations?hl=en&user=rZgHBHcAAAAJ) |
| [Dangayach NS](https://pubmed.ncbi.nlm.nih.gov/?term=Dangayach+NS&size=200) |
